# List of Reviewers

**DOI:** 10.1017/S0031182022001743

**Published:** 2023-05

*Parasitology* would like to thank the following people, who kindly provided reviews for the journal in 2022:

- Tyler Achatz, Middle Georgia State University, United States
- Coen Adema, University of New Mexico, United States
- Poom Adisakwattana, Mahidol University Faculty of Tropical Medicine, Thailand
- Karim Tarik Adjou, ANSES, United Kingdom of Great Britain and Northern Ireland
- Edson Adriano, Universidade Federal de São Paulo, Brazil
- Maria Aguirre-Macedo, CINVESTAV IPN, Mexico
- Ehsan Ahmadpour, Tabriz University of Medical Sciences, Iran (the Islamic Republic of)
- Luciana M. R. Alencar, Univ Fed Maranhao, United Kingdom of Great Britain and Northern Ireland
- Silmara Allegretti, UNICAMP - Universidade Estadual de Campinas, Brazil
- Sonia Almeria, US Food and Drug Administration, United States
- Abdulaziz Alouffi, King Abdulaziz City for Science and Technology, Saudi Arabia
- Leucio Alves, Federal Rural University of Pernambuco (UFRPE), Brazil
- Philippe Alves, Universidade Federal de Minas Gerais, Brazil
- Márcia Attias, Universidade Federal do Rio de Janeiro, Brazil
- Carlos Azevedo, Institute of Biomedical Sciences Abel Salazar, University of Porto, Portugal
- Ivana BILIC, University of Veterinary Medicine, Austria
- Simon Babayan, University of Glasgow, United Kingdom of Great Britain and Northern Ireland
- Nathan Bahr, University of Kansas Medical Center, United States
- Anna Bajer, University of Warsaw, Poland
- Eric Bakwo Fils, University of Maroua, Cameroon
- Paulius Baltrusis, Swedish Univ Agr Sci, United Kingdom of Great Britain and Northern Ireland
- Iain Barber, Nottingham Trent University, United Kingdom of Great Britain and Northern Ireland
- Daniel Bargieri, University of Sao Paulo, Brazil
- Kathryn Bartley, Moredun Research Institute, United Kingdom of Great Britain and Northern Ireland
- Diane Barton, Charles Sturt University - Wagga Wagga Campus, Australia
- Paul Bates, Lancaster University, United Kingdom of Great Britain and Northern Ireland
- jolanta Behnke-Borowczyk, Poznan University of Life Sciences, Poland
- Chloe Berger, Laval University, Canada
- Heloisa Bertagnon, Universidade Estadual do Centro-Oeste, Brazil
- Brown Besier, Brown Besier Parasitology Pty Ltd, Australia
- Ian Beveridge, University of melbourne, Australia
- David Blair, James Cook University, Australia
- Jamie Bojko, Teesside University, United Kingdom of Great Britain and Northern Ireland
- Tiffany Bouchery, Swiss Tropical and Public Health Institute, Switzerland
- Alexandre Bougdour, IAB - Centre de Recherche Inserm U1209 / CNRS UMR5309 / UGA, United States
- Dwight Bowman, Cornell, United States
- Paola Braicovich, Universidad Nacional de Mar del Plata, Argentina
- Annely Brandt, Landesbetrieb Landwirtschaft Hessen, Germany
- Sara Brant, University of New Mexico, United States
- Rod Bray, Natural History Museum, United Kingdom of Great Britain and Northern Ireland
- Emanuele Brianti, University of Messina, Italy
- Timea Brázová, Institute of Parasitology of the Slovak Academy of Sciences, Slovakia
- kurt Buchmann, University of Copenhagen, Denmark
- Joanne Cable, Cardiff University, United Kingdom of Great Britain and Northern Ireland
- Janine Caira, University of Connecticut, United States
- Rafael Calero-Bernal, Complutense University of Madrid, Spain
- Cinzia Cantacessi, Cambridge Veterinary School, United Kingdom of Great Britain and Northern Ireland
- Chris (Katharine C.) Carter, Strathclyde University, United Kingdom of Great Britain and Northern Ireland
- Francisco Castaño Vázquez, National Museum of Natural Sciences, Spain
- Gabriela Certad, Institut Pasteur de Lille, France
- Carolina Chagas, Nature Research Centre, Lithuania
- Jong-Yil Chai, Seoul National University College of Medicine, Korea (the Republic of)
- Nayden Chakarov, Bielefeld University, Germany
- Leslie Chisholm, University of Adelaide Faculty of Sciences, Australia
- Georgios Christodoulopoulos, University of Thessaly, Greece
- Lavinia Ciuca, University of Naples Federico II, Italy
- Nicholas Clark, The University of Queensland, Australia
- Christopher Coates, Swansea University, United Kingdom of Great Britain and Northern Ireland
- Eduardo Coelho, Universidade Federal de Minas Gerais, Brazil
- Gerald Coles, Retired, United Kingdom of Great Britain and Northern Ireland
- James Cotton, University of Glasgow College of Medical Veterinary and Life Sciences, United Kingdom of Great Britain and Northern Ireland
- Kevin Couper, University of Manchester, United Kingdom of Great Britain and Northern Ireland
- Thomas Crellen, University of Oxford Big Data Institute, United Kingdom of Great Britain and Northern Ireland
- Thomas Cribb, University of Queensland, Australia
- Stefano D'Amelio, University of Rome, Italy
- John Dalton, National University of Ireland Galway, Ireland
- Arwid Daugschies, Faculty of Veterinary Medicine, Germany
- Andrew David, Clarkson University, United States
- Claudio De Liberato, Istituto Zooprofilattico Sperimentale delle Regioni Lazio e Toscana, Italy
- Wanderley De Souza, INSTITUTO DE BIOFÍSICA CARLOS CHAGAS FILHO, Brazil
- Victor Del Rio-Araiza, Universidad Nacional Autonoma de Mexico, Mexico
- Paul Denny, Durham University, United Kingdom of Great Britain and Northern Ireland
- Yves Desdevises, Universite Pierre et Marie Curie - Paris 6, France
- Sheila Donnelly, University of Technology, Sydney, Australia
- Mert Doskaya, Ege University Faculty of Medicine, Turkey
- Regina Draghi, Universidad Nacional de la Plata, Argentina
- Hongxia Duan, Chinese Acad Sci, China
- Anna Dubiec, Polish Academy of Sciences, Poland
- Altangerel Dursahinhan, University of Nebraska State Museum and School of Biological Sciences, United States
- Daniela Dutra, Instituto de Ciências Biológicas, Universidade Federal de Minas Gerais, Brazil
- Alvaro Díaz, Universidad de la Republica., Uruguay
- Ramon Eichenberger, Universitat Zurich, Switzerland
- Tamara Emmenegger, Lund University, United Kingdom of Great Britain and Northern Ireland
- S Espuelas, Tropical health Institute, Spain
- Jorge Falcon, Universidad Autónoma del Estado de Hidalgo, Mexico
- Ted Fan, Taipei Medical University, Taiwan
- Alan Fecchio, CONICET-CIEMEP, Argentina
- Valeria Felix de Lima, UNESP, Brazil
- Ivan Fiala, Biological Centre, AS CR, Czech Republic
- Vlatka Filipović Marijić, Institut Ruđer Bošković, Croatia
- Maria Fioravanti, University of Bologna, Italy
- Pier Luigi Fiori, University of Sassari, Italy
- Jerome Follet, Institut Supérieur D'agriculture, France
- Sarah Gabriel, University of Ghent, Belgium
- Kirill Galaktionov, Zoological Institute RAS, Russian Federation
- Luz Garcia-Longoria Batanete, University of Extremadura, Spain
- Gilles Gargala, Rouen Institute for Research and Innovation in Biomedicine, France
- Thomas Gasan, Queen's University Belfaast, United Kingdom of Great Britain and Northern Ireland
- Umut Gazi, Near East University, Turkey
- Stefan Geiger, Universidade Federal de Minas Gerais, Brazil
- Peter Geldhof, Ghent University, Belgium
- Matt Gibbins, University of Glasgow, United Kingdom of Great Britain and Northern Ireland
- Wendy Gibson, Bristol University, United Kingdom of Great Britain and Northern Ireland
- David Gibson, The Natural History Museum, United Kingdom of Great Britain and Northern Ireland
- John Gilleard, University of Calgary, Canada
- Mathieu Gissot, Institut Pasteur de Lille, France
- Itamar Glazer, Agricultural Research Organization Volcani Center, Israel
- Henrik Glenner, University of Bergen, Norway
- Suchi Goel, Indian Institute of Science Education and Research, India
- Luis Gomez-Puerta, Universidad Nacional Mayor de San Marcos, Peru
- M. Gonzalez, Universidad de Antofagasta, Chile
- Javier González Miguel, Institute of Natural Resources and Agrobiology of Salamanca, Spain
- Manish Goyal, Hebrew University of Jerusalem Faculty of Science, Israel
- Carlos Graeff-Teixeira, Universidade Federal do Espírito Santo, Brazil
- Stephen Greiman, Georgia Southern University, United States
- Edmundo Grisard, Universidade Federal de Santa Catarina (UFSC), Brazil
- Ricardo Gurtler, University of Buenos Aires, Argentina
- Jorge Gutiérrez, University of Extremadura, Spain
- Wilfried Haas, University Erlangen-Nuernberg, Germany
- Julius Hafalla, LSHTM, United Kingdom of Great Britain and Northern Ireland
- Patrick Hamilton, University of Exeter, United Kingdom of Great Britain and Northern Ireland
- Kelly Hayes, The University of Manchester, United Kingdom of Great Britain and Northern Ireland
- Felix Heckendorn, FIBL, Switzerland
- David Heins, Tulane University, United States
- Andrew Hemphill, University of Bern, Switzerland
- Jesús Hernández-Orts, Univeristy of Valencia, Czech Republic
- Paulo Hofstatter, Campinas State University, Brazil
- Emily Hotchkiss, University of Glasgow School of Veterinary Medicine, United Kingdom of Great Britain and Northern Ireland
- Kuo-Yang Huang, National Defense Medical Center, Taiwan
- Sandra Inacio, Universidade Estadual Paulista Júlio de Mesquita Filho, Brazil
- CARLOS JORGE RAMIREZ FLORES, University of Wisconsin-Madison, United States
- Susan Jarvi, University of Hawai'i at Hilo, United States
- Franz Jirsa, University of Vienna, Austria
- Pikka Jokelainen, Statens Serum Institut, Denmark
- Malcolm Jones, The University of Queensland, Australia
- Alexandra Juhász, University of Veterinary Medicine, Hungary
- Nadira Karunaweera, University of Colombo Faculty of Medicine, Sri Lanka
- Frank Katzer, Moredun Research Institute, United Kingdom of Great Britain and Northern Ireland
- Jane Kelley, Australia Department of Primary Industries and Energy, Australia
- Ulrike Kemmerling, Universidad de Chile, Chile
- Anna Kildemoes, Leiden University Medical Center, Netherlands
- Dean Kinjevic, University of Zagreb Faculty of Veterinary Medicine, Croatia
- Hans Klompen, Ohio State University, United States
- Yoon Kong, Sungkyunkwan University, Korea (the Republic of)
- Aneta Kostadinova, Central Laboratory of General Ecology, Bulgarian Academy of Sciences, Bulgaria
- Ivica Kralova-Hromadova, Slovak Academy of Sciences, Slovakia
- Boris Krasnov, Jacob Blaustein Institutes for Desert Research, Ben-Gurion University of the Negev, Israel
- Roman Kuchta, Biology Centre Czech Academy of Sciences Institute of Parasitology, Czech Republic
- Roman Kuchta, Biology Centre, Academy of Sciences of the Czech Republic, Institute of Parasitology, Czech Republic
- Katrin Kuhls, Technische Fachhochschule Wildau, Germany
- K.J. Senthil Kumar, National Chung Hsing University, Taiwan
- Stephen Larcombe, The University of Edinburgh, United Kingdom of Great Britain and Northern Ireland
- Marcela Lareschi, CEPAVE (CCT- La Plata, CONICET-UNLP), Argentina
- Maria Latrofa, University of Bari, Italy
- Scott Lawton, Scotland's Rural College - Inverness Campus, United Kingdom of Great Britain and Northern Ireland
- Hoang Le, Skretting Aquaculture Research Centre, Norway
- Matthieu Le Bailly, University of Bourgogne Franche-Comte, France
- David Leitsch, Medical University of Vienna, Austria
- Liang Li, Hebei Normal University, China
- Dieter Liebhart, Clinic for Avian, Reptile and Fish Medicine, Austria
- Tim Littlewood, Natural History Museum, United Kingdom of Great Britain and Northern Ireland
- Fan Liu, Xiamen Univ, United Kingdom of Great Britain and Northern Ireland
- Sam Loker, University of New Mexico, United States
- Alex Loukas, James Cook University, Australia
- Jan Lovy, N.J. Division of Fish & Wildlife, United States
- Andrew MacColl, University of Nottingham, United Kingdom of Great Britain and Northern Ireland
- Ewan MacLeod, The University of Edinburgh, United Kingdom of Great Britain and Northern Ireland
- J MacRae, The Francis Crick Institute, United Kingdom of Great Britain and Northern Ireland
- Luis Madeira de Carvalho, University of Lisbon, Portugal
- Rick Maizels, University of Glasgow, United Kingdom of Great Britain and Northern Ireland
- Ben Makepeace, University of Liverpool, United Kingdom of Great Britain and Northern Ireland
- Wanchai Maleewong, Faculty of Medicine, Khon Kaen University, Thailand
- David Marcogliese, Environment and Climate Change Canada Québec, Canada
- Jose Alejandro Martinez Ibarra, Universidad de Guadalajara, Mexico
- Santiago Mas-Coma, Universidad de Valencia, Spain
- Alexander Mastin, APHA, United Kingdom of Great Britain and Northern Ireland
- Sonja Matthee, Stellenbosch University, South Africa
- Simonetta Mattiucci, “Sapienza” University of Rome, Italy
- Maria Paola Maurelli, University of Naples Federico II, Italy
- Sergei Medvedev, Zoologiceskij institut RAN, Russian Federation
- Heinz Mehlhorn, Department of Parasitology, United Kingdom of Great Britain and Northern Ireland
- Jelena Melesina, Martin-Luther-Universitat Halle-Wittenberg, Germany
- Jairo Alfonso Mendoza-Roldan, University of Bari, Valenzano, Italy, Italy
- Roberto Luis Mera y Sierra, Juan Agustin Maza University, United Kingdom of Great Britain and Northern Ireland
- Santiago Merino, Museo Nacional de Ciencias Naturales, Spain
- Victor Midlej, Universidade Federal do Rio de Janeiro, Brazil
- Andrei Mihalca, University of Agricultural Sciences and Veterinary Medicine Cluj-Napoca, Romania
- Hamed Mirjalali, Shahid Beheshti University of Medical Sciences Research Institute for Gastroenterology and Liver Diseases, Iran (the Islamic Republic of)
- Piers Mitchell, University of Cambridge, United Kingdom of Great Britain and Northern Ireland
- Tapoka Mkandawire, The Francis Crick Institute, United Kingdom of Great Britain and Northern Ireland
- Wessam Mohamed Ahmed Mohamed, Hokkaido Univ, United Kingdom of Great Britain and Northern Ireland
- Scott Monks, Universidad Autonoma del Estado de Hidalgo Centro de Investigaciones Biologicas, Mexico
- Serge Morand, CNRS, France
- Frantisek Moravec, Institute of Parasitology, Biology Centre of the Czech Academy of Science, Czech Republic
- Rodrigo Morchón, University of Salamanca, Spain
- Viatcheslav Mordvinov, Institute of Cytology and Genetics SB RAS, Russian Federation
- Samson Mukaratirwa, University of KwaZulu-Natal, South Africa
- Alfonso Navas, Museo Nacional de Ciencias Naturales, CSIC, Spain
- Chris Niebuhr, Landcare Research New Zealand, New Zealand
- Harry Noyes, University of Liverpool, United Kingdom of Great Britain and Northern Ireland
- Jahashi Nzalawahe, Sokoine University of Agriculture, Tanzania, United Republic of
- Lúcia O' Dwyer, Universidade Estadual Paulista, Brazil
- Kazuo Ogawa, Meguro Parasitology Musuem, Japan
- B Okamura, Natural History Museum, United Kingdom of Great Britain and Northern Ireland
- Antti Oksanen, Finnish Food Authority, Finland
- Peter Olson, The Natural History Museum, United Kingdom of Great Britain and Northern Ireland
- Marcello Otake Sato, Dokkyo Medical University, Japan
- Mirna Oviedo, Universidad Técnica de Manabí, Ecuador
- Francesc Padrós, Autonomous University of Barcelona, Spain
- Maria Pakharukova, Institute of Cytology and Genetics SB RAS, Russian Federation
- Jose Palacios-Abella, University of Valencia Cavanilles Institute of Biodiversity and Evolutionary Biology, Spain
- Oswaldo Palenzuela, Inst. Aquaculture (CSIC), Spain
- Lais Pardini, Facultad de Cs Veterinarias, Argentina
- Eunji Park, University of Otago Department of Zoology, New Zealand
- Rachel Paterson, Norwegian Institute for Nature Research, Norway
- Josephine Pegg, South African Institute for Aquatic Biodiversity, South Africa
- Tom Pennance, Natural History Museum London, United Kingdom of Great Britain and Northern Ireland
- Susan Perkins, American Museum of Natural History, United States
- Marie-Jeanne Perrot-Minnot, Université de Bourgogne, France
- Romualda Petkevičiūtė, Nature Research Centre, Lithuania
- Enrique Peñalver, Instituto Geológico y Minero de España, Spain
- Stephen Phillips, University of Glasgow, United Kingdom of Great Britain and Northern Ireland
- Hudson Pinto, Universidade Federal de Minas Gerais, Brazil
- Priscila Pinto, Federal University of Juiz de For a, Brazil
- Veronica Poggio, Instituto de Ciencia y Tecnologia Cesar Milstein, Argentina
- Robert Poulin, University of Otago, New Zealand
- Eduardo Pozio, Istituto Superiore di Sanità, Italy
- Satarudra Prakash Singh, Mahatma Gandhi Central University, India
- Whitney Preisser, University of Washington, United States
- Bronwen Presswell, University of Otago New Zealand., United Kingdom of Great Britain and Northern Ireland
- Gerardo Pérez-Ponce de León, Universidad Nacional Autónoma de México, Mexico
- Petra Quillfeldt, Justus Liebig University Giessen, Germany
- Rupert Quinnell, Leeds University, United Kingdom of Great Britain and Northern Ireland
- Yvonne Qvarnstrom, Centers for Disease Control and Prevention, United States
- R Rae, Liverpool John Moores University, United Kingdom of Great Britain and Northern Ireland
- Thomas Raffel, Oakland University, United States
- Angel Ramos, Universidad Veracruzana, Mexico
- Lisa Ranford-Cartwright, University of Glasgow, United Kingdom of Great Britain and Northern Ireland
- Carlos Rauque, National University of Comahue, Argentina
- Susanne Reier, Naturhistorisches Museum Wien, Austria
- Karl Reinhard, School of Natural Resource Sciences University of Nebraska-Lincoln, United Kingdom of Great Britain and Northern Ireland
- Philip Riekenberg, NIOZ, Netherlands
- Thierry Rigaud, Universite de bourgogne, France
- Nichol Ripley, University of Kentucky, United States
- Koert Ritmeijer, Medecins Sans Frontieres, Netherlands
- Mark Robinson, Queens University Belfast, United Kingdom of Great Britain and Northern Ireland
- Alicia Rojas, University of Costa Rica | UCR Centro de Investigación en Enfermedades Tropicales, Costa Rica
- Bruce Rosa, Washington University School of Medicine in Saint Louis, United States
- Heinz Sager, Elanco Animal Health Inc., Switzerland
- Guillaume Sallé, INRAE, UMR 1282 Infectiologie et Santé Publique, France
- Juliana Sanchez, Universidad Nacional del Noroeste de la Provincia de Buenos Aires, Argentina
- Antoine Sanou, Centre National de Recherche et de Formation sur le Paludisme, Burkina Faso
- Diego Santiago-Alarcon, University of South Florida College of Arts & Sciences, United States
- Cláudia Santos, Instituto Oswaldo Cruz, Brazil
- Bahador Sarkari, Shiraz University of Medical Sciences, Iran (the Islamic Republic of)
- Yukita Sato, Nihon University, Japan
- Joseph Schall, University of Vermont, United States
- Achim Schnaufer, The University of Edinburgh, United Kingdom of Great Britain and Northern Ireland
- Gabriele Schonian, Charite University Medicine Berlin, Germany
- Rolf K. Schuster, Central Veterinary Research Laboratory, United Arab Emirates
- W. Evan Secor, CDC, United Kingdom of Great Britain and Northern Ireland
- Aleksandra Sedzikowska, Medical University of Warsaw, Poland
- Shookofeh Shamsi, Charles Sturt University, Faculty of Science, Wagga Wagga, Australia
- David Shapiro, USDA-ARS SEA, United States
- Andy Shinn, INVE (Thailand) Ltd, Benchmark, Thailand
- Kgomotso Sibeko-Matjila, University of Pretoria, South Africa
- Andrea Simkova, Faculty of Science, Masaryk University, Czech Republic
- Anthony Sinai, University of Kentucky, United States
- L.D. Singla, Guru Angad Dev Veterinary and Animal Sciences University,
- Paiboon Sithithaworn, Khon Kaen University, Thailand
- Victoria Sleight, University of Aberdeen, United Kingdom of Great Britain and Northern Ireland
- Nico Smit, North West University, South Africa
- Tom Smith, Swiss tropical Institute, Switzerland
- Adrian Smith, University of Oxford, United Kingdom of Great Britain and Northern Ireland
- Matthew Smith, USGS, United States
- Gabriele Sorci, Univeristé de Bourgogne, France
- Javier Sotillo, Carlos III Health Institute, Spain
- Banchob Sripa, Khon Kaen University, Thailand
- Michal Stanko, Slovak Academy of Sciences, Slovakia
- Alexandr Stekolnikov, Zoological Institute, Russian Federation
- J. Stothard, Liverpool School of Tropical Medicine, United Kingdom of Great Britain and Northern Ireland
- Sutas Suttiprapa, Khon Kaen University Faculty of Medicine, Thailand
- Andrew Sweet, Arkansas State University, United States
- Jan Tachezy, Charles University, Czech Republic
- Christina Tadiri, McGill University, Canada
- Sirikachorn Tangkawattana, Khon Kaen University, Thailand
- Jouni Taskinen, University of Jyväskylä, Finland, Finland
- Urusa Thaenkham, Mahidol University Faculty of Tropical Medicine, Thailand
- Alexandre Thibodeau, University of Montreal, Canada
- David Thieltges, Royal Netherlands Institute for Sea Research (NIOZ), Netherlands
- Juan Timi, Universidad Nacional de Mar del Plata Facultad de Ciencias Exactas y Naturales, Argentina
- Rafael Toledo, Facultad de Farmacia-Universidad de Valencia, Spain
- Timir Tripathi, North-Eastern Hill University, India
- Takafumi Tsuboi, Ehime University, Japan
- Troy Tuckey, William & Mary Virginia Institute of Marine Science, United States
- Kevin Tyler, UEA, United Kingdom of Great Britain and Northern Ireland
- Hanif Ullah, Shanghai Veterinary Research Institute Chinese Academy of Agricultural Sciences, China
- Aleksandra Uzelac, Institute for Medical Research, Serbia
- Ana Valente, Universidade Federal de Pelotas, Brazil
- Antonio Varcasia, Università degli Studi di Sassari, Italy
- Grace Vaziri, University of Connecticut, United States
- Jozef Vercruysse, University of Ghent. Laboratory of Parasitology, Faculty of Veterinary Medicine, Belgium
- Jaco Verweij, Elisabeth-TweeSteden Ziekenhuis, Netherlands
- Victor Vidal-Martinez, Centro de Investigación y de Estudios Avanzados del Instituto Politécnico Nacional, Mexico
- Marina Vinaud, UFG, Brazil
- Hannah Vineer, University of Liverpool Faculty of Science, United Kingdom of Great Britain and Northern Ireland
- Mark Viney, University of Bristol, United Kingdom of Great Britain and Northern Ireland
- Antonio Vázquez, Pedro Kouri Institute of Tropical Medicine Center for Diagnostic and Reference Research, Cuba
- Heather Walden, University of Florida, United States
- Richard Wall, University of Bristol, United Kingdom of Great Britain and Northern Ireland
- Chun-Ren Wang, Heilongjiang Bayi Agricultural University, China
- Lesley Warner, South Australian Museum, Australia
- Bonnie Webster, Natural History Museum, United Kingdom of Great Britain and Northern Ireland
- Christopher Whipps, SUNY College of Environmental Science and Forestry, United States
- Christopher Williams, Liverpool John Moores University, United Kingdom of Great Britain and Northern Ireland
- R Wilson, University of York Department of Biology, United Kingdom of Great Britain and Northern Ireland
- Radosław Włodarczyk, University of Lodz Faculty of Biology and Earth Sciences, Poland
- Ting-Bao Yang, Center for Parasitic Organisms，State Key Laboratory of Biocontrol, School of Life Sciences, Key Laboratory for Tropical Diseases Control of the Ministry of Education, China
- Nigel Yarlett, Pace University, United States
- Alparslan Yildirim, Erciyes Universitesi, Turkey
- Shigeto Yoshida, Kanazawa University, Japan
- Vyacheslav Yurchenko, University of Ostrava, Czech Republic
- Qin-Ping Zhao, Wuhan University, China
- Annetta Zintl, University College Dublin, Ireland
- Xavier de Montaudouin, University of Bordeaux, France
- george poinar, oregon state university, United States
- Barbora Červená, University of Veterinary and Pharmaceutical Sciences Brno, Czech Republic
- Pavel Široký, University of Veterinary and Pharmaceutical Sciences, Faculty of Veterinary Hygiene and Ecology, Czech Republic
- Marta Špakulová, Parasitological Institute SAS, Slovakia
- Rita Žiegytė, Nature Research Centre, Lithuania

